# A Statistical Model for Regional Tornado Climate Studies

**DOI:** 10.1371/journal.pone.0131876

**Published:** 2015-08-05

**Authors:** Thomas H. Jagger, James B. Elsner, Holly M. Widen

**Affiliations:** Department of Geography, Florida State University, Tallahassee, Florida, United States of America; Medical University of Vienna, AUSTRIA

## Abstract

Tornado reports are locally rare, often clustered, and of variable quality making it difficult to use them directly to describe regional tornado climatology. Here a statistical model is demonstrated that overcomes some of these difficulties and produces a smoothed regional-scale climatology of tornado occurrences. The model is applied to data aggregated at the level of counties. These data include annual population, annual tornado counts and an index of terrain roughness. The model has a term to capture the smoothed frequency relative to the state average. The model is used to examine whether terrain roughness is related to tornado frequency and whether there are differences in tornado activity by County Warning Area (CWA). A key finding is that tornado reports increase by 13% for a two-fold increase in population across Kansas after accounting for improvements in rating procedures. Independent of this relationship, tornadoes have been increasing at an annual rate of 1.9%. Another finding is the pattern of correlated residuals showing more Kansas tornadoes in a corridor of counties running roughly north to south across the west central part of the state consistent with the dryline climatology. The model is significantly improved by adding terrain roughness. The effect amounts to an 18% reduction in the number of tornadoes for every ten meter increase in elevation standard deviation. The model indicates that tornadoes are 51% more likely to occur in counties served by the CWAs of DDC and GID than elsewhere in the state. Flexibility of the model is illustrated by fitting it to data from Illinois, Mississippi, South Dakota, and Ohio.

## Introduction

Broad-scale tornado climatology in the United States is well described and physically understood. The seasonal spread of the tornado threat from the deep South northward into the northern Plains and Midwest during summer is tied to the poleward migration of the jetstream [1]. A concentration of tornado activity across Oklahoma and Kansas during spring is linked to the vertical intersection of mid-level dry air from the Rockies and abundant low-level moist air from the Gulf of Mexico [2].

Regional-scale tornado climatology is less well described and poorly understood. One reason for this is because tornadoes are discrete events, spatially clustered, and locally quite rare. Another reason for this is because of the uneven quality of the tornado record [3, 4]. While the U.S. tornado database is the largest in the world, it contains issues that limit its utility for climate studies [5]. For instance, improved observation practices have led to an increase in the reporting of weak tornadoes [6, 7]. Even today many weak, short duration tornadoes likely go undocumented in places with few people or poor communication infrastructure. This observational effect is well known [5, 8] although it appears to have diminished during the most recent decade [9].

Various methods for quantifying and modeling the observational effects have been proposed [10–12]. Most studies assume a uniform region of activity and estimate tornado frequency within a subset of the region likely to be most accurate. The uniform regions are defined by the available data. Tornado reports are often aggregated using kernel smoothing [13–15]. Spatial density maps that show regions of higher and lower tornado frequency are useful for exploratory analysis and hypothesis generation but because there is no simple way to control for environmental or other factors, interpretation of the patterns can be misleading. Another drawback is the implicit assumption that tornado occurrences are independent. This is generally not the case as a single supercell thunderstorm can generate a family of tornadoes [16].

This research asks the question; how can regional tornado climatology be recovered from a heterogeneous database of rare, clustered events? The question is answered with a statistical model that produces a map of smoothed tornado occurrence that reflects regional patterns of physical forcing. The available data are first aggregated to the county level. Aggregation makes it easy to include human and environmental data (population, terrain, percent agriculture, etc.) to control for known effects in the data. The model is fit using the method of integrated nested Laplacian approximation (INLA) to solve the Bayesian integrals arising from the application of Bayes theorem. This setup accommodates non-normally distributed counts and correlated residuals. The correlated residuals (random-effects term) shows where tornado activity is high relative to the state average. The method described in this paper is valuable because it has the potential to uncover the remaining spatial patterns of tornado activity after controlling for selected covariates.

Data preparation and modeling procedures are described first for Kansas. The procedures are then demonstrated for Illinois, Mississippi, South Dakota, and Ohio representing different tornado-prone areas in the United States. For each state, an index of terrain roughness is tested to see whether it improves the model fit. It is reasonable to assume that terrain roughness might be influential in some areas but not others. For instance, terrain roughness might be less important in areas with large variations in land use and land cover than in areas of the Great Plains. In addition, the National Weather Service (NWS) County Warning Areas (CWA) are used to identify areas with significantly higher and lower tornado rates.

The balance of the paper is outlined as follows. The tornado database and identified reporting issues are described in section 2. The tornado report frequency by Kansas county is evaluated in section 3. The statistical model used to estimate tornado occurrence by county while controlling for non-physical factors is described in section 4 and the results from fitting the model to tornado reports first from Kansas then from Illinois, Mississippi, South Dakota, and Ohio are shown in section 5. The influence of terrain roughness on tornado frequency conditional on the model is examined in section 6. In section 7, key findings are summarized and suggestions made for future work.

## Materials and Methods

### Data

#### Boundaries, elevation, and population

**Fig 1:**
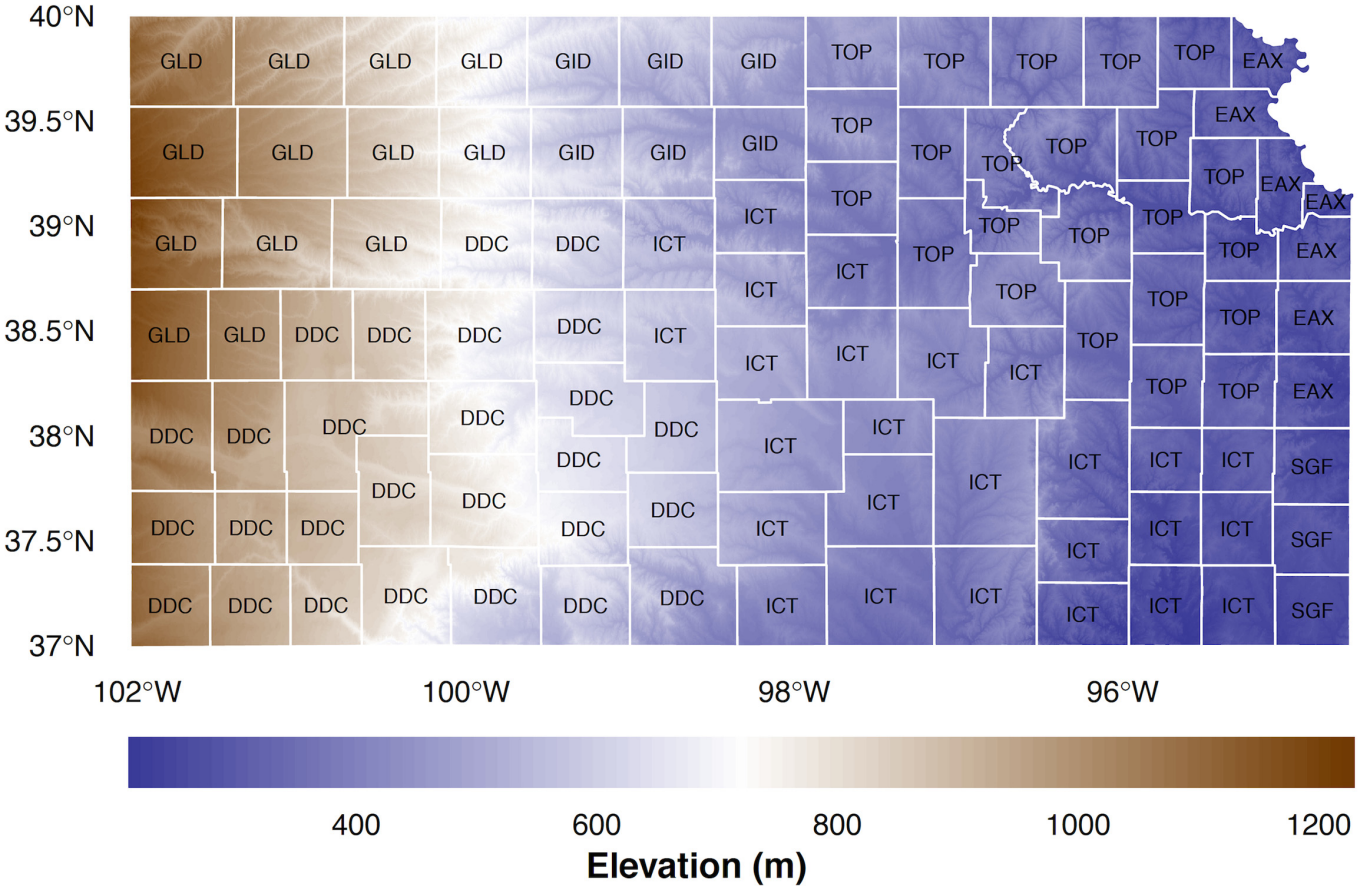
Kansas counties and elevation. Counties are labeled by the corresponding CWA. Elevation is given at a resolution of 80 m.

The model is written with the open-source R language using freely-available government data including tornadoes from the U.S. Storm Prediction Center (SPC), population and administrative boundaries from the U.S. Census Bureau, and elevations from NASA’s Shuttle Radar Topography Mission (SRTM). The data are prepared as follows. First county administrative boundaries for the United States are downloaded and read into R as vector polygons from https://www.census.gov/geo/maps-data/data/cbf/cbf_counties.html at a resolution of 1:5 million and subset by the state of interest using the Federal Information Processing Standard (FIPS) code. Then digital elevation model (DEM) data are downloaded from http://www.viewfinderpanoramas.org at a resolution of three arc seconds (approximately 80 m) and read into R as a raster. The elevation raster is cropped to the state boundary. Next CWA labels from http://www.nws.noaa.gov/geodata/catalog/wsom/data/bp03de14.dbx are attached to each county. The results for Kansas are displayed on a map in Fig 1.

Elevation (above mean sea level) ranges from less than 220 m in the east to higher than 1220 m in the west. The Kansas River in the northeast and its tributaries extending westward are visible at this spatial resolution. These elevations are used to compute an index of terrain roughness. The three-letter abbreviation of the corresponding CWA is given in each county. The CWAs include Dodge City (DDC), Goodland (GLD), Topeka (TOP), Wichita (ICT), North Platte (LBF), Omaha/Valley (OAX), Hastings (GID), and Kansas City/Pleasant Hill (EAX). The DDC NWS is responsible for 27 Kansas counties followed by 26 for ICT and 23 for TOP.

**Fig 2:**
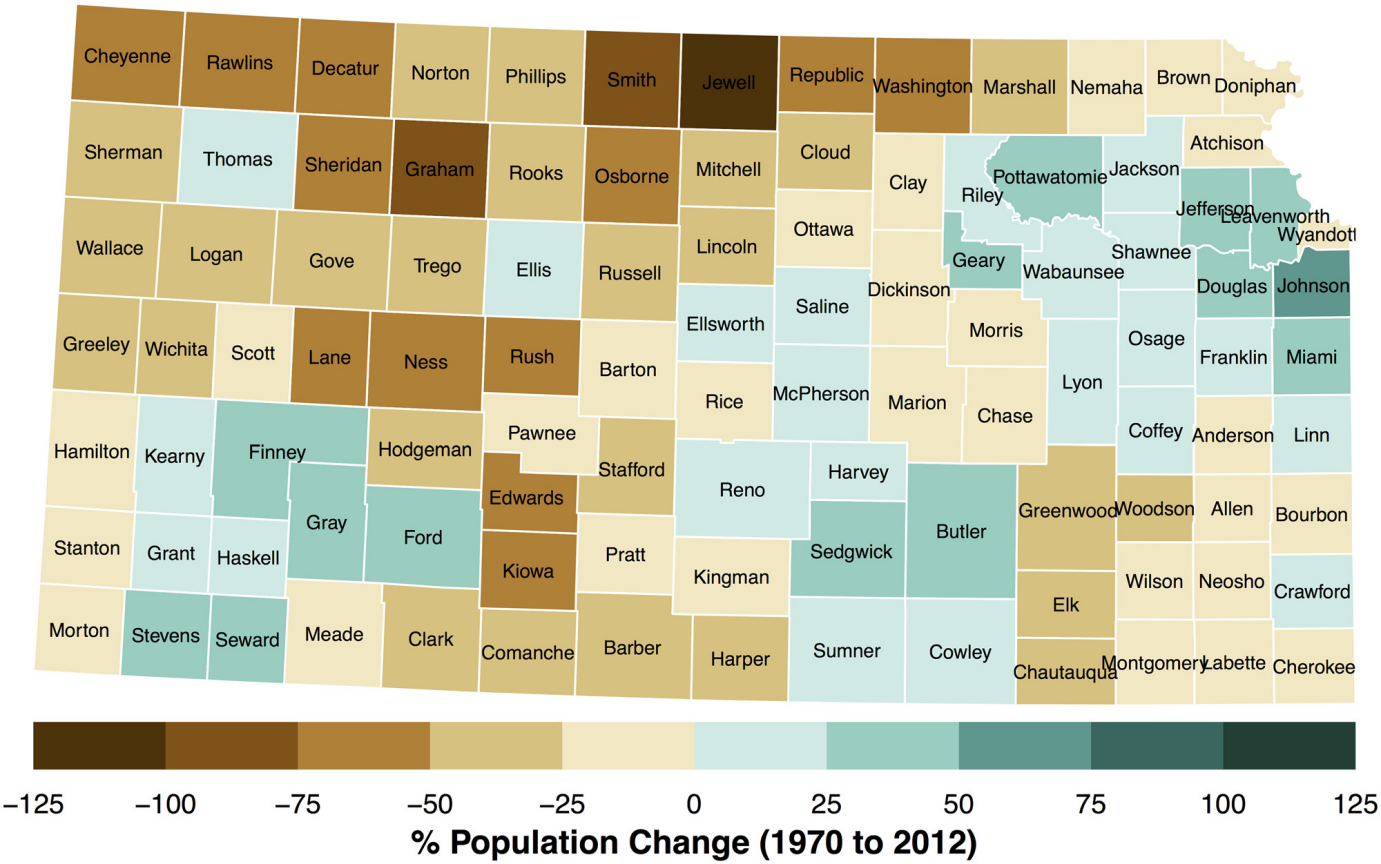
Population changes between 1970 and 2012. The change is expressed as a percentage difference with 2012 as the base year.

**Fig 3:**
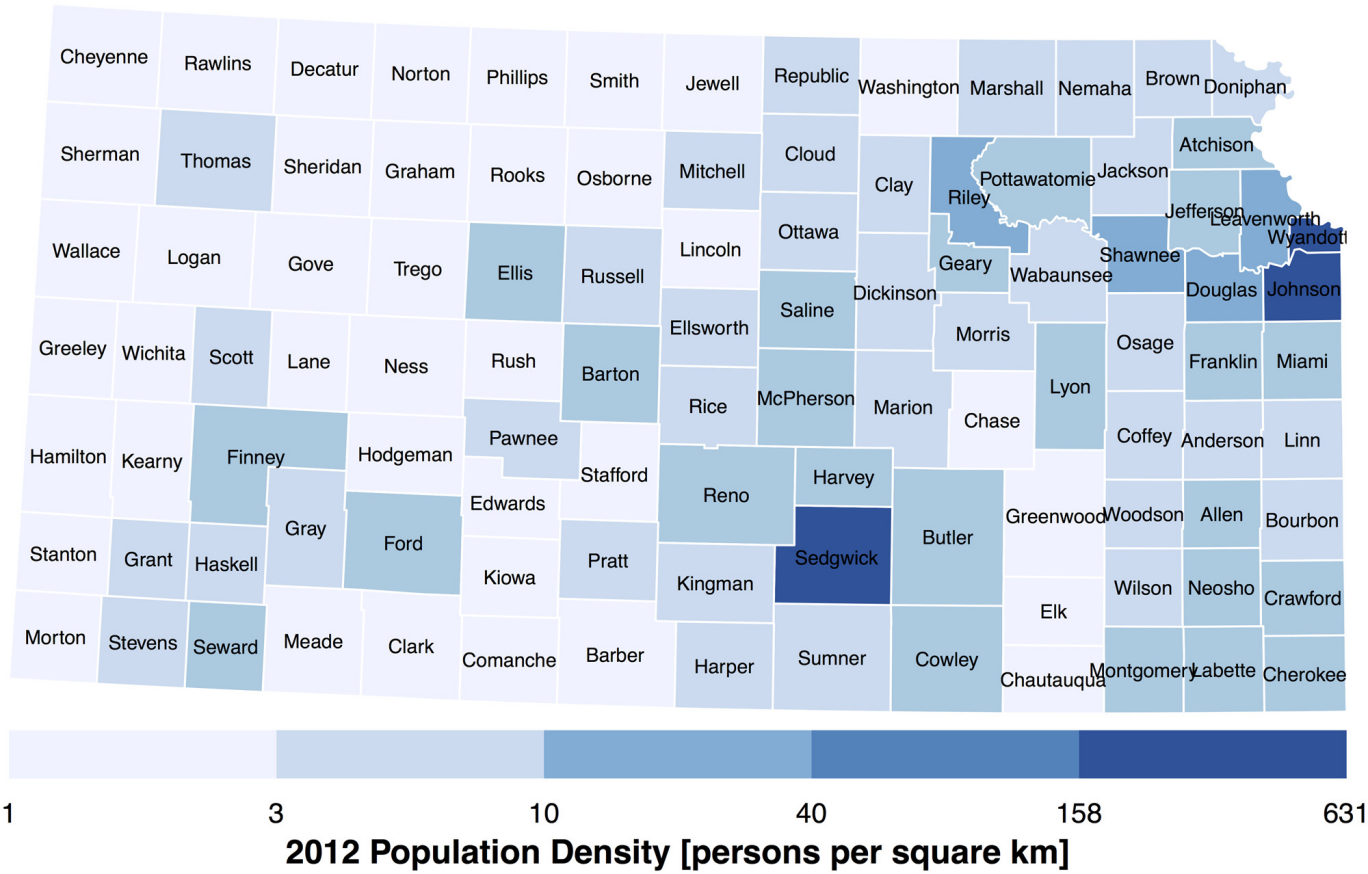
Population estimates for 2012 by county. Values are expressed as persons per square km.

Data preparation continues by adding annual population estimates over the period 1970–2013 from http://www.nber.org/data/census-intercensal-county-population.html to each county. The percentage change over this period using 2012 as the baseline is displayed on a Lambert conformal conic map in Fig 2. Counties in blue indicate more people in 2012 compared to 1970. Counties to the south and west of Kansas City show the largest increases. Butler and Sedgwick counties (Wichita area) and Ford, Gray, and Finney (Dodge City area) also show large percentage increases although the latter area has fewer people (Fig 3). Population densities exceeding 190 people per square kilometer are found in Wyandotte (Kansas City), Johnson, and Sedgwick counties. Population densities for 2013 are estimated using the 2012 county values.

#### Tornado Tracks

Next the SPC database containing all reported tornadoes in the United States over the period 1950–2013 is obtained from www.spc.noaa.gov/gis/svrgis/zipped/tornado.zip. Individual reports in the database are compiled by the NWS offices and reviewed by the National Climate Data Center [6]. The database comes in a shapefile format with each tornado provided as a straight line track. Tornado information in the database is considered reliable for climate studies [17]. The tornado track is the great circle line (no width) between the estimated start (touchdown) and end locations. Locations are recorded with two digit decimal precision prior to 2009 and four digit afterwards. Locations are more accurate later in the record when estimates are made with GPS. Not all tornadoes tracks are in a straight line nor do they all remain in contact with the ground along the entire path. No attempt is made to adjust for possible variations from a continuous straight line track.

Tornado reports tend to be more numerous near cities compared to rural areas but this spatial variation is decreasing with time [9]. Moreover, improvements in observational practices tend to result in a larger number of tornado reports, especially reports of weak tornadoes [6, 18]. Tornadoes are rated on a damage scale from 0 (least) to 5 [19, 20], with the earliest tornadoes in the database rated retroactively [12, 21, 22]. To improve the precision on the ratings the Enhanced Fujita Scale, which includes more damage indicators, was adopted in 2007 [23]. Changes to population and to the rating procedures result in a heterogeneous database. Consistent with advice given by the SPC [6] our analysis is limited to tornadoes that occurred from 1970 through 2013 rated EF1 and higher on the damage scale. In this paper, the word ‘tornado’ refers to tornadoes that received a damage rating of at least EF1. County tornado counts are accumulated for each tornado track that falls within or that crosses into the county during each year.

**Fig 4:**
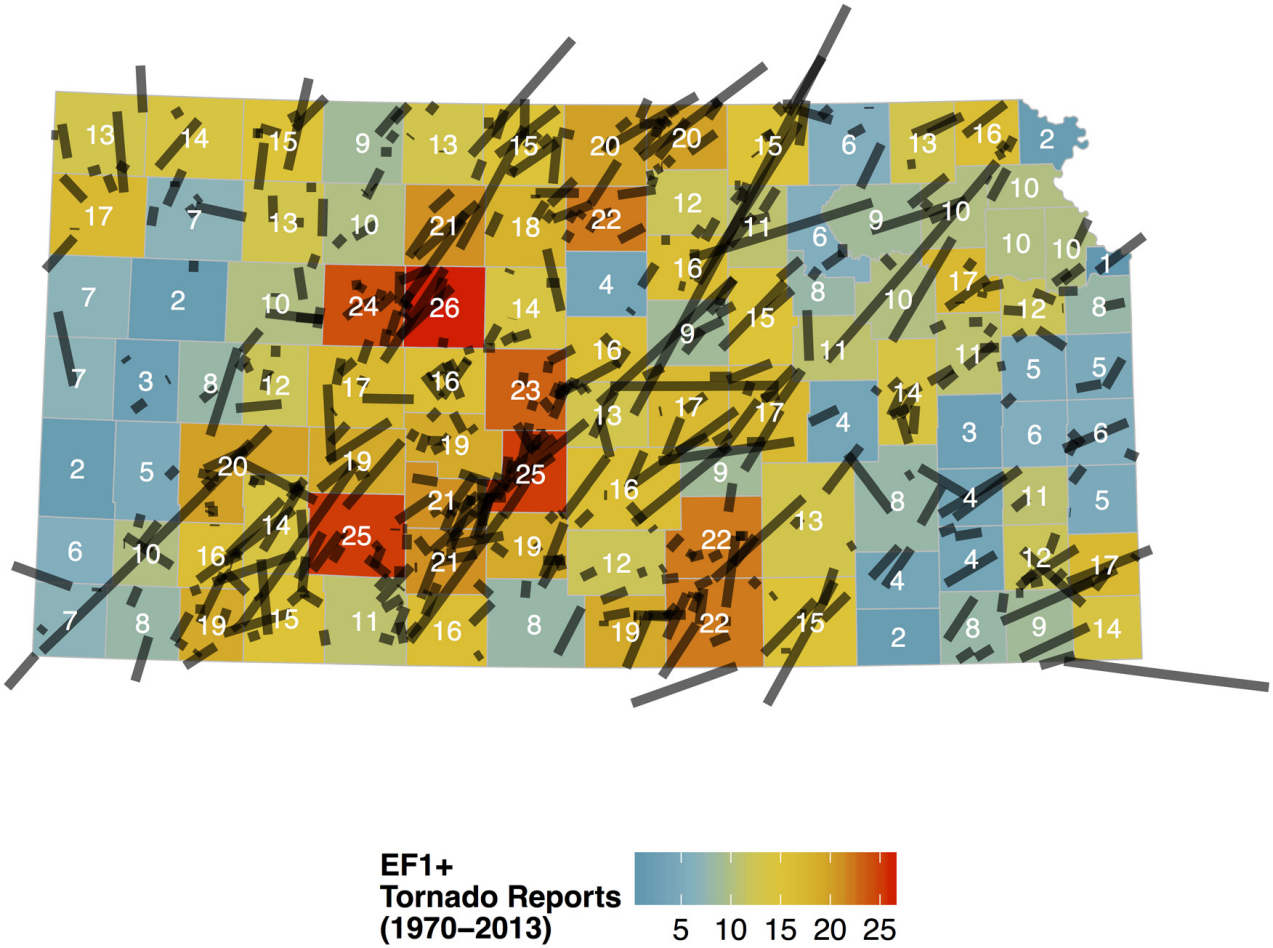
Tornado report frequency by county for Kansas. Only tornadoes rated EF1 and higher are used. Lines show the tornado track. The shortest tracks are not visible at this scale. Total tornado counts over the period 1970–2013 are listed inside the county and the color scale is from few (blue) to many (red).

**Fig 5:**
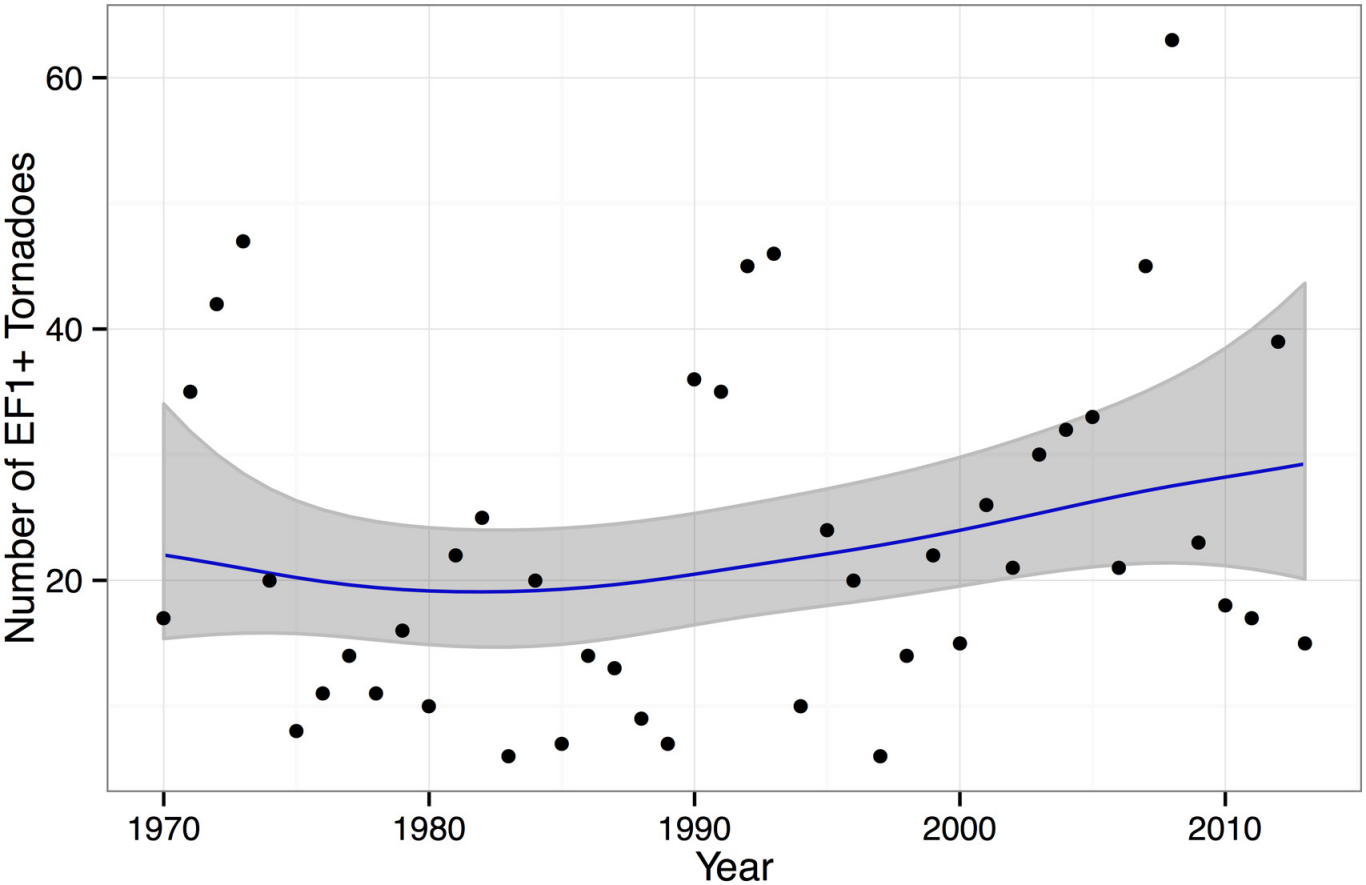
Statewide tornado counts for Kansas from 1970–2013. The trend line uses a second-order random walk model where the counts are described by a negative binomial distribution. The 90% uncertainty band is shown in gray.

The result is a space-time data set with constant-time attributes that include county area and terrain roughness, and variable-time attributes that include the annual number of tornadoes and population density. Area is converted to units of square kilometers and the tornado rate per county is computed as the number of tornadoes per 10,000 square kilometers per year. Tornadoes are most numerous across central Kansas (Fig 4). The larger counties tend to have more tornado reports although the relationship is not large [*r* = .34 (.19, .48) 90% CI] since the counties tend to have similar sizes. Regional hot spots include Sedgwick County (city of Wichita) and parts of the northeast in the counties around Kansas City. The correlation between the 2012 county population and the number of tornadoes is not significant [*r* = .04 (−.12, .20) 90% CI]. The annual number of tornado reports for the state as a whole has increased since 1970 at a rate of less than one per year, but the trend is not significant (Fig 5). Summary statistics are listed in Table 1.

**Table 1:** Summary of the data analysis and modeling results. DIC is the deviance information criterion, AD is the Anderson-Darling test, and *r* is the Pearson correlation coefficient.

	Kansas	Illinois	Mississippi	South Dakota	Ohio
FIPS	20	17	28	46	39
No. counties	105	102	82	66	88
Area (km^2^)	210,845	144,451	123,701	199,367	105,954
Avg Elevation [m]	580.9 (580.5,581.2)	189.1 (189.0,189.2)	85.70 (85.62,85.78)	665.0 (664.6,665.4)	279.6 (279.5,279.7)
No. tornadoes (nT) [EF1+]	1010	879	1112	423	501
*r*(Area, nT)	.34 (.19,.48)	.64 (.53,.72)	.55 (.41,.67)	−.11 (−.30,.10)	.34 (.17,.49)
Single tornado most counties	7	8	12	3	6
Population [2012]	2,893,957	12,882,135	2,991,207	833,354	11,544,225
*r*(Population [2012], nT)	.04 (−.12,.20)	.14 (−.02,.30)	.49 (.34,.62)	.39 (.20,.55)	.20 (.03,.37)
Tornado trend [%/yr]	.87 (−.27,2.0)	.48 (−.77,1.75)	.44 (−.78,1.67)	−1.60 (−3.04,−.14)	−1.45 (−2.85,−.03)
DIC (w/out spatial term)	6027	5268	5729	2544	3364
DIC (w/ spatial term)	5990	5211	5680	2500	3302
AD *p* value	>.15	>.15	>.15	>.15	>.15
Log score	.635	.568	.770	.448	.436
Brier score	.570	.415	.564	.269	.212
Pop density term	.1187 (.0655,.1723)	.1083 (.0525,.1643)	.1304 (.0734,.1868)	.1693 (.0791,.2569)	.0714 (−.0051,.1466)
Trend term	.0189 (.0054,.0323)	n.s.	.0230 (.0039,.0422)	−.0173 (−.0318,−.0029)	n.s.
Interaction term	−.0045 (−.0073,−.0017)	−.0016 (−.0036,.0004)	−.0050 (−.0083,−.0018)	n.s.	−.0031 (−.0053,−.0010)
*r*(Roughness, nT)	−.067 (−.256,.126)	−.173 (−.355,.022)	−.023 (−.239,.195)	−.115 (−.347,.131)	−.066 (−.271,.146)
DIC (w/ Roughness term)	5980	5212	5678	2502	3303
Roughness term	−.0186 (−.0268,−.0106)	−.0051 (−.0173,.0073)	−.0098 (−.0194,−.0003)	−.0020 (−.0039,.0000)	.0003 (−.0126,.0133)
DIC (w/ CWA term)	5981	5213	5669	2881	3352

### Method

The main idea of this paper is to demonstrate a model for tornado occurrence at the county level. The model is useful for climate studies because it includes a term that captures the smoothed frequency relative to the state average after accounting for known non-climate factors. To account for changes in tornado reporting due to population shifts over time the log_2_ annual county population density is included as a fixed-effect term. Further, to account for improvements in rating procedures over time, the calendar year and an interaction term of year with log_2_ population density are also included as fixed-effect terms. Finally, to account year to year changes, a random effect term is added.

Inferences on the number of tornadoes in each county, *s* for each year *t*, *T*_*s*,*t*_ is assumed to be adequately described by a negative binomial distribution with parameters probability *p* and size *r*. Elsner and Widen (2014) [24] show that the negative binomial distribution better describes tornado count data than does a Poisson distribution. If *X* is a random sample from this distribution, then the probability that *X* = *k* is $P(k|r,p)=\left(\begin{array}{c} k+r-1\\ k \end{array}\right){\left(1-p\right)}^{r}p^{k}$, for *k* ∈ 0, …, ∞, *p* ∈ (0,1) and *r* > 0. This relates the probability of observing *k* successes before the *r* failure of a series of independent events with probability of success equal to *p*.

The distribution is generalized by allowing *r* to be any positive real number and it arises from a Poisson distribution whose rate parameter has a gamma distribution. Whereas the Poisson distribution has a variance equal to its mean, the negative binomial distribution is over dispersed. This means that the ratio of the variance to the average exceeds one which implies that the underlying process generating the counts is clustered. To simplify the process of making inferences, the distribution is re-formulated using the mean $\mu =r\frac{p}{1-p}$ and the size *r* to allow a separation of the mean effect from the dispersion parameter.

The mean of the negative binomial distribution, *μ*_*s*,*t*_ is linked to a linear combination of the predictors and random effects, *ν*_*s*,*t*_ through the exponential function and the area of the county, *A*_*s*_. The dispersion is modeled with a scaled size parameter *n* where *n* = *r*_*s*,*t*_/*A*_*s*_ giving a dispersion of 1/*p*_*s*,*t*_ = 1 + μ_*s*,*t*_/*n* = 1 + exp(ν_*s*,*t*_)/*n* that depends only on the tornado density and *n*. To keep *n* small, the area of each county in square km is divided by 2000.

More concisely the model is:

$$\begin{array}{ccc} T_{s,t}|\mu_{s,t},r_{s,t} & ∼ & \text{NegBin}(\mu_{s,t},r_{s,t})\\ \mu_{s,t} & = & A_{s}\exp\left(\nu_{s,t}\right)\\ \nu_{s,t} & = & \beta_{0}+\beta_{1}\;{\text{lpd}}_{s,t}+\beta_{2}\;\left(t-t_{0}\right)+\beta_{3}\;{\text{lpd}}_{s,t}\left(t-t_{0}\right)+u_{s}+v_{t}\\ r_{s,t} & = & A_{s}\;n \end{array}$$

where NegBin(*μ*_*s*,*t*_, *r*_*s*,*t*_) indicates that the conditional tornado counts (*T*_*s*,*t*_∣*μ*_*s*,*t*_,*r*_*s*,*t*_) are described by a negative binomial distribution with mean *μ*_*s*,*t*_ and size *r*_*s*,*t*_, lpd_*s*,*t*_ represents the base 2 logarithm of the annual population density for each region, and *t*_0_ is the base year set to 1991 (middle year of the record).

The correlated spatial random effects term *u*_*s*_ follows an intrinsic Besag formulation with the sum-to-zero constraint [25]:

$$\begin{array}{ccc} u_{i}|\left\{u_{j,j\neq i},\tau \right\} & ∼ & N(\frac{1}{m_{i}}\sum_{i∼j}u_{j},\frac{1}{m_{i}}\tau )\\ \sum_{\forall i}u_{i} & = & 0 \end{array}$$

where *N* is the normal distribution with mean 1/*m*_*i*_ · ∑_*i* ∼ *j*_ *u*_*j*_ and variance 1/*m*_*i*_ · 1/*τ* where *m*_*i*_ is the number of neighbors of county *i* and *τ* is the precision; *i* ∼ *j* indicates the two counties *i* and *j* are neighbors. Neighboring counties are determined by contiguity (queen’s rule) using functions from the **spdep** package [26]. The annual uncorrelated random effect, *v*_*t*_, is modeled as a sequence of normally distributed random variables, with mean 0 and variance 1/*τ*′

The prior on the vector of spatial random effects is statistically independent from the vector of annual random effects. For each posterior sample, the vector of spatial random effects has the same values for all years and the vector of annual random effects has the same values for all regions as implied by the subscripts in the model notation. Gaussian priors with low precision are assigned to the *β*’s. To complete the model the scaled size (*n*) is assigned a log-gamma prior and the precision parameters (*τ* and *τ*′ are assigned a log-Gaussian prior. Although yet to be used on county-level tornado data, a similar model was recently constructed for modeling hurricane data [27] and these types models are frequently used for mapping disease rates [28, 29].

The priors and the likelihood are combined using Bayes rule to obtain the posterior distributions for the model parameters. Since the integrals cannot be solved analytically, a common technique is to use a Markov chain Monte Carlo (MCMC) algorithm to obtain samples from the posterior distributions. Here the method of integrated nested Laplace approximation (INLA) is used instead. INLA provides a fast alternative for models with a latent Gaussian structure [30] and is accomplished with functions from the **INLA** package [31].

The model fits the data well. The probability integral transform (PIT) values modified for small counts are adequately described by a uniform distribution [32]. The adequacy is checked by noting that the *p*-value on an Anderson-Darling (AD) goodness-of-fit test under the null hypothesis of a uniform distribution exceeds .15. The predictive quality of the model is assessed by the cross-validated log score. A smaller value of the score indicates better predictive quality [33]. The log score is.635 for Kansas, which is better than the log scores for seasonal tornado models [24]. The Brier score is .570 as the mean squared difference between the predicted probability and the actual count in each county for each year (105 × 45 = 4725 predictions). The Brier score for the null model is .603.

The coefficient on the logarithm (base 2) of population density has a posterior mean of .1187 [(.0655, .1723) 90% credible interval (CI)] (Table 1). This translates to a 13% [(exp(.1187) − 1) × 100%] increase for a doubling of the population. The coefficient on the year (trend) term has a posterior mean of.0189 [(.0054, .0323) 90% CI]; statistically significant and upward at a rate of 1.9% per year. The interaction term is also statistically significant with a posterior mean of −.0045, indicating a decrease in the influence of population density. In fact, the model indicates that the influence of population density on the tornado reports will approach zero by the year 2017 [*β*_1_ + *β*_3_ (2017 − 1991) ≈ 0], although practically there will likely continue to be at least some influence of population on the reports for some time to come.

### Correlated random effects

**Fig 6:**
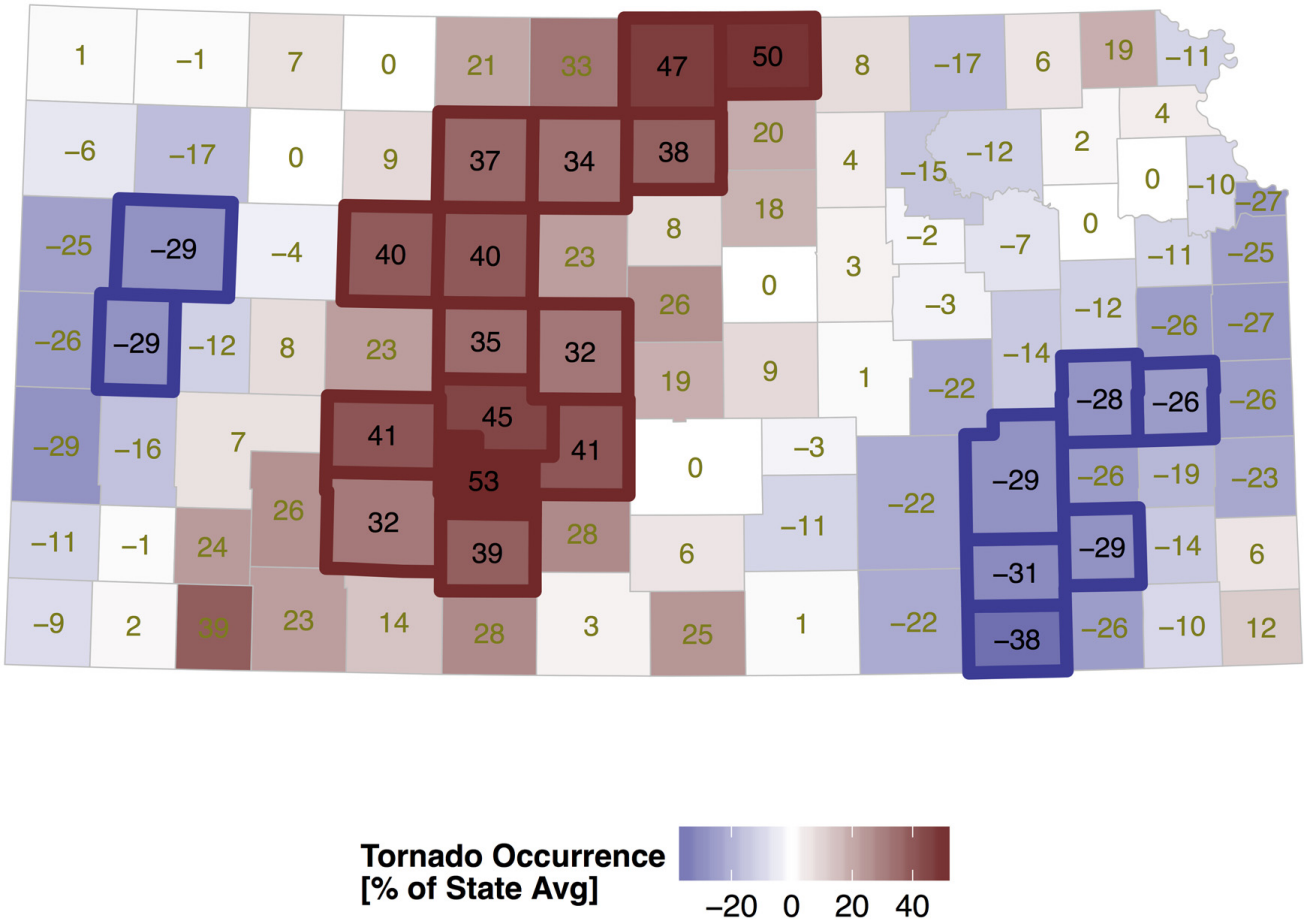
Correlated random effects from the Kansas tornado model. Values are the posterior mean and are expressed as the percent difference from the state average. The model includes annual population density and calendar year as fixed effects.

**Fig 7:**
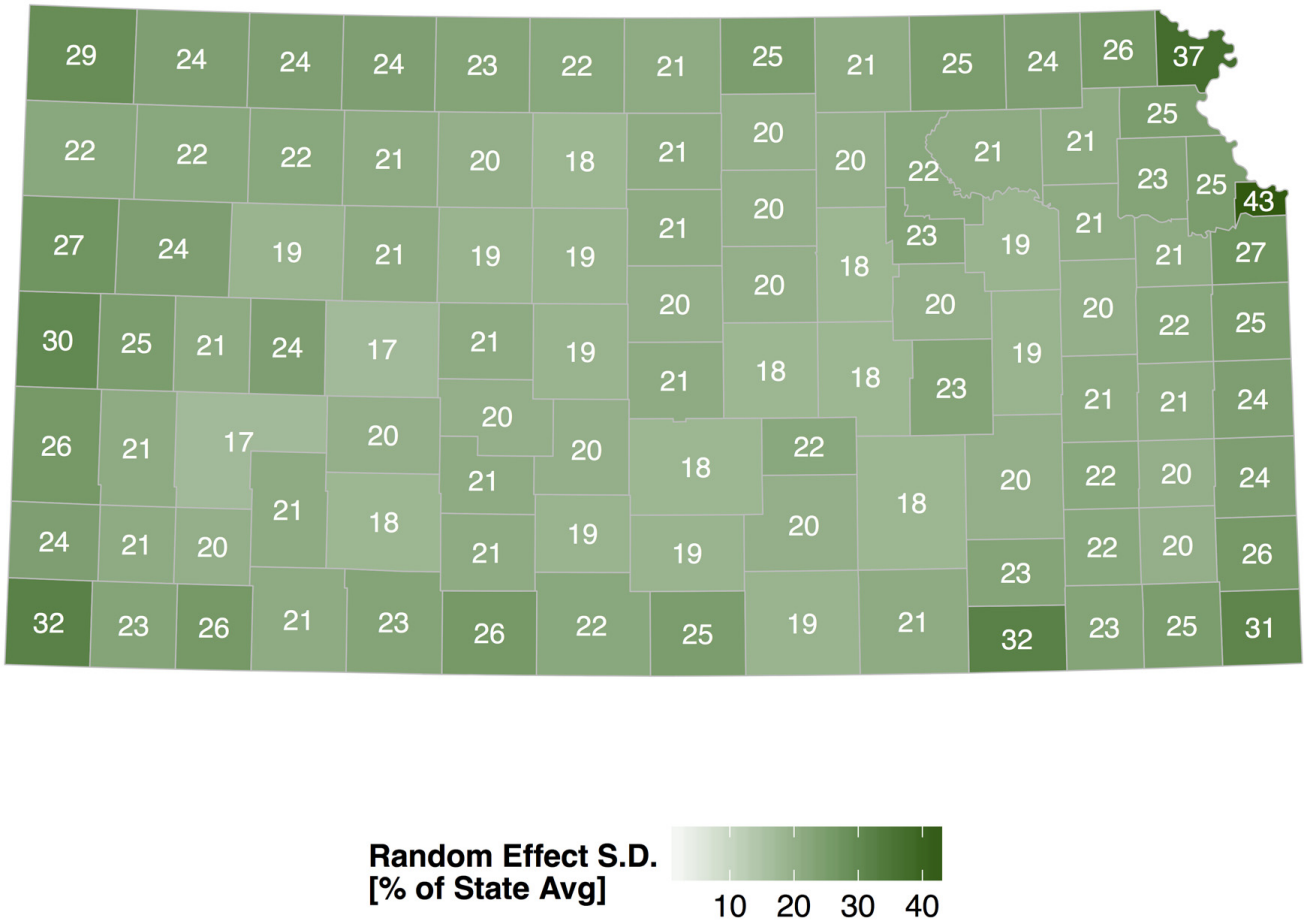
Standard deviation of the correlated random effects from the Kansas tornado model. Values have units of percent difference from the state average.

The random-effects term is the spatially correlated set of residuals that provides a description of tornado occurrence statewide that accounts for population changes, differences in exposure, and trend within each county. A map of this term reveals where tornadoes are more likely relative to the state average (Fig 6) after controlling for population density, county area and annual variation. Values are the posterior means and are expressed as a percent difference from the state average. Counties with significantly (at the 90% level) higher and lower rates are outlined in bold. Uncertainty on the magnitude of these values is measured by the posterior standard deviation (Fig 7). Standard deviations tend to be lower (precision higher) in counties with more neighbors (away from the state borders).

The map features a north-south axis of above-average activity across the west central part of the state with lower activity to the west (as found in [13]) and generally lower activity to the east. The axis of above-average activity in the north is shifted somewhat farther to the east. The four counties of Hodgeman, Edwards, Pawnee, and Stafford in south central Kansas have tornado activity that exceeds the average by at least 40% as do Jewell and Republic counties in the north.

Nearly three quarters of Kansas tornadoes occur from April through June. Surface low pressure in eastern Colorado to the lee of the Rockies in response to westerly winds aloft produce veering southeasterly surface winds across the state. These winds transport moisture up slope (Fig 1) with deep convection initiating in western Kansas along the dryline. The dryline forms in the High Plains during spring and separates moist air originating over the Gulf of Mexico from dry air originating over the southwestern United States and high plateau of Mexico [34]. Initial thunderstorm organization results in discrete supercells east of the dryline along a roughly north-south axis. The discrete cells tend to merge into a mesoscale convective system across eastern Kansas after sunset reducing the threat for tornadoes. Strong winds, heavy rains, and frequent lightning become the main concern to life and property.

### Index of terrain roughness as a fixed effect

Next the model is used to test whether terrain roughness can help explain the pattern of tornadoes across Kansas. The test is motivated by the physical hypothesis that a tornado is somewhat more likely to occur, all else being equal, where the low-level inflow is unimpeded. Studies have shown that surface roughness affects this inflow; in particular it affects the velocity distribution, pressure distribution, and the core radius of the flow [35–38]. An increase in terrain roughness causes the maximum tangential velocity to decrease [38]. But experimental studies have argued that the roughness used in these studies are outside the range of values encountered in nature [39].

Here the standard deviation in the 80-m resolution elevation data is computed within each county and used as a proxy for terrain roughness. Counties with smaller elevation standard deviations are smoother. Values range from a low of 11.3 m to 73.4 m with the smoother counties in the southeast part of the state. The model is refit using terrain roughness as an additional fixed effect. The DIC decreases to 5980 indicating a better model with this term included (Table 1). Elevation itself is not a significant term when included in the model.

**Fig 8:**
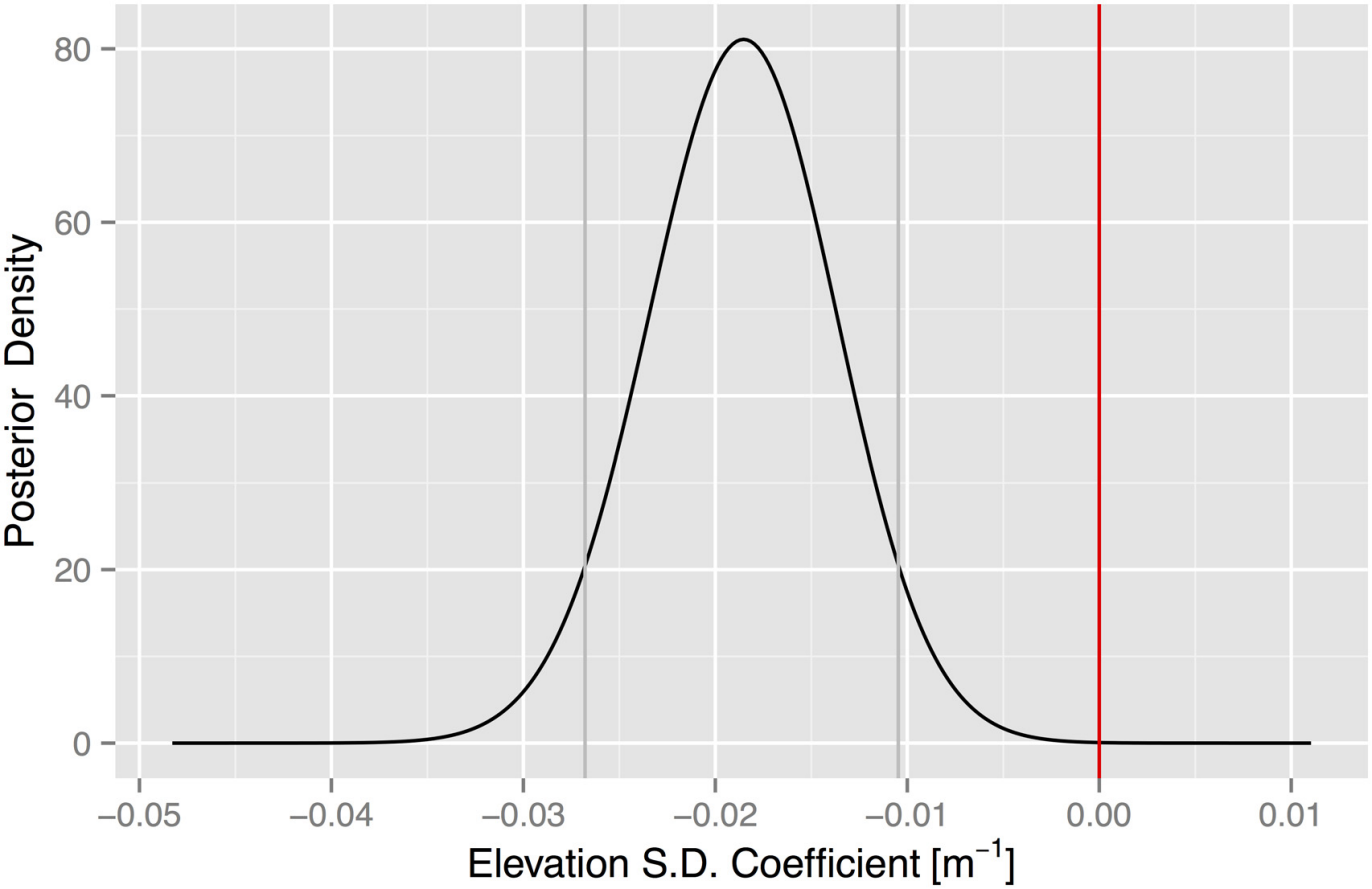
Posterior density of the elevation standard deviation term. The 90% CI is shown with the vertical gray lines. The red line indicates no effect.

The magnitude of the effect is indicated by the size of the coefficient. The posterior mean of the coefficient is −.0186 [(−.0268, −.0106) 90% CI] indicating an 18% reduction in the tornado occurrence for every ten meter increase in elevation standard deviation. The significance of the effect is indicated with a plot of the posterior density (Fig 8). The density is offset to the left of zero, where zero indicates the proxy for terrain roughness has no relationship to tornadoes at the county level.

This finding is consistent with [40] who show a negative relationship between the occurrence location of tornadoes and elevation variance. However, [40] consider only touchdown locations of intense (EF3+) tornadoes and a domain that covers the eastern two-thirds of the United States. They also use a coarser (approximately 1 km) elevation database. Instead of the standard deviation in elevation, we use the difference between the lowest and highest elevation as a proxy for terrain roughness and find a similar effect (not shown).

**Fig 9:**
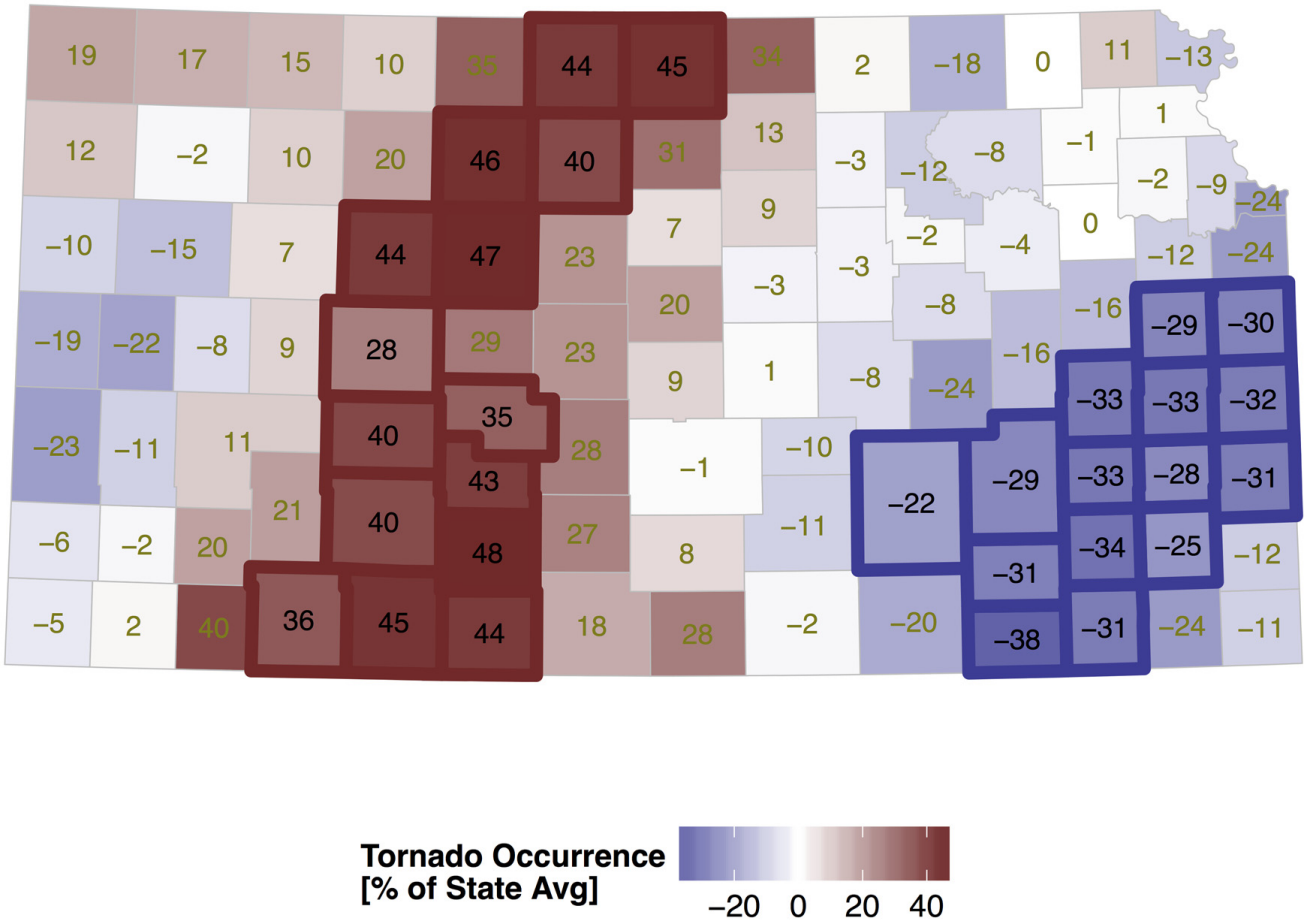
Same as Fig 6 except the model has elevation standard deviation as an additional fixed effect.

Since the roughness term is significant, it is added to the model and the correlated random-effects term re-evaluated (Fig 9). The overall pattern remains unchanged with a corridor of enhanced activity across the west-central part of the state. This example shows how to test hypotheses concerning factors that could be related to tornado activity by representing the values at the county-level and included the term in the model.

### County Warning Area as a fixed effect

Next the model is used to check whether there are significant variations in tornado activity by CWA. Variations do not necessarily imply different warning and verification practices. Nevertheless to improve consistency across offices it is instructive to know whether more attention to variations is warranted. The CWA term is treated as a factor variable where each county is given the name of the corresponding CWA (see Fig 1). The term is included as a fixed effect. The DIC with this term increases to 5981 showing that the model with the CWA is less likely to replicate the observed distribution of tornado activity than the same model without the CWA. However, when the DDC CWA (Dodge City, KS) and the GID CWA (Grand Island, NE) are included as a single combined binary variable (DDC and GID or neither) the DIC drops to 5977. The coefficient on the binary term is.4112 [(.2185, .6011) 90% CI] indicating that tornadoes are 51% more likely to have occurred in counties served by these two CWAs than elsewhere in the state. The DDC and GID offices are responsible for warnings across central Kansas where tornadoes tend to be most numerous and the spatial random effect is mostly positive.

### Illinois, Mississippi, South Dakota, and Ohio

**Fig 10:**
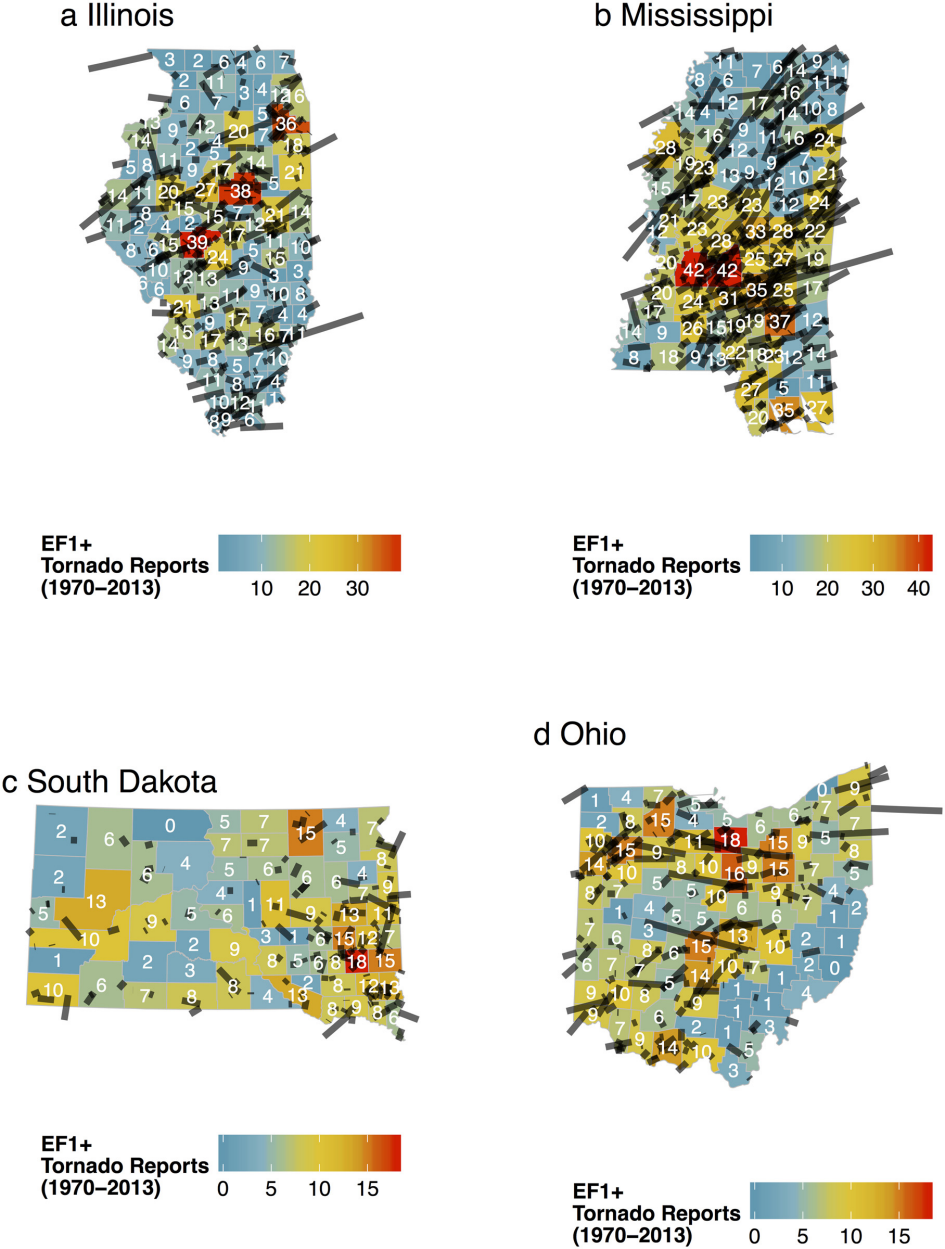
Tornado report frequency by county for Illinois, Mississippi, South Dakota, and Ohio.

Flexibility of the model is demonstrated by fitting it to tornado and environmental data from four additional states including Illinois, Mississippi, South Dakota, and Ohio. The choice of states is based on a representative sample of other tornado-prone areas in the United States. The summary and model statistics discussed below are listed by state in Table 1. Maps of raw tornado counts by county for the four states are shown in Fig 10. The procedures for preparing the data at the county level are the same as before. An exception occurs for South Dakota where an additional raster of elevations is needed for counties north of 45° N latitude. Like across Kansas, tornado counts are significantly correlated with county size in Illinois, Mississippi, and Ohio. South Dakota is the exception where the larger counties in the western half of the state tend to have fewer tornadoes compared to the smaller counties in the southeast corner.

**Fig 11:**
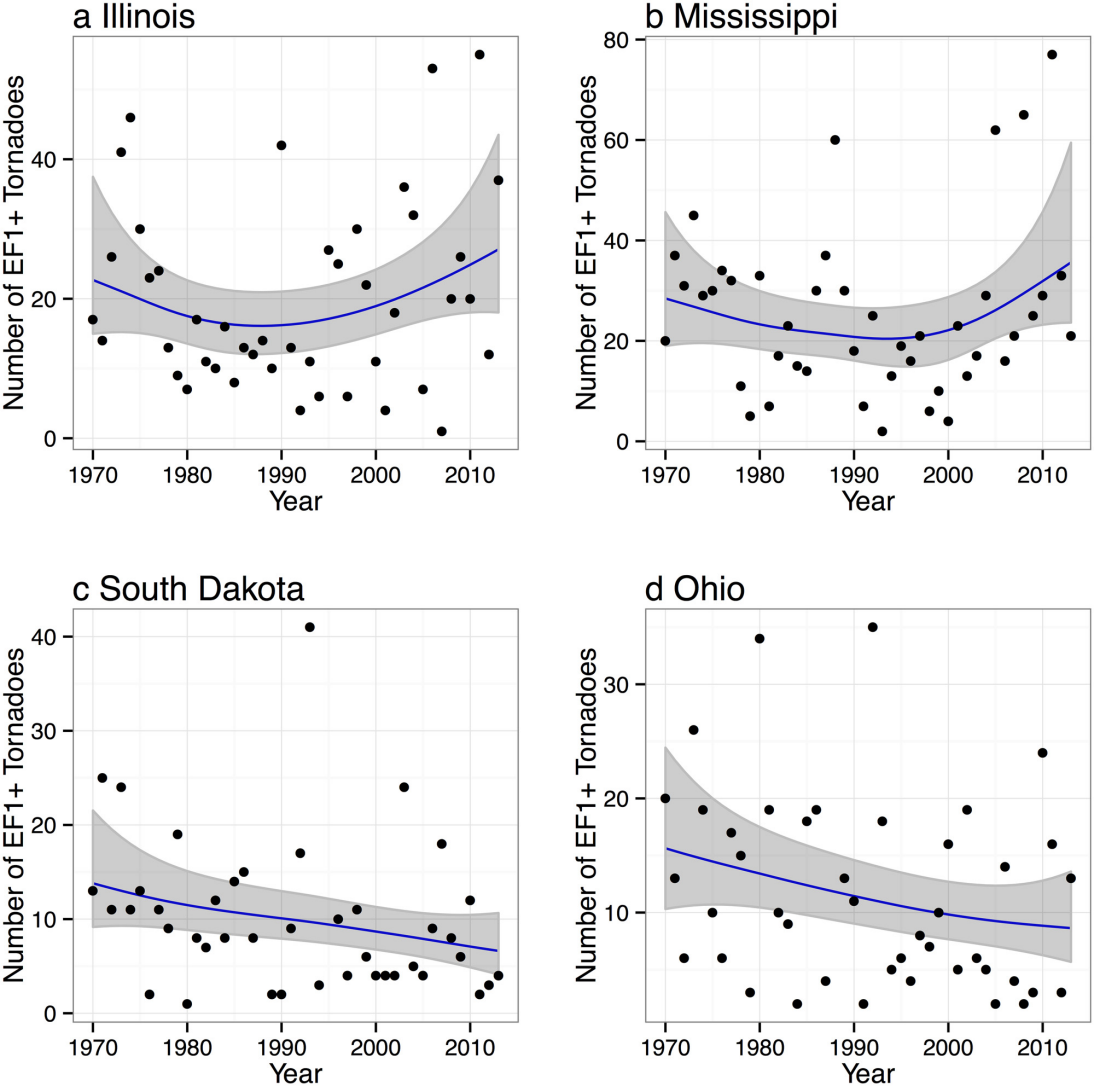
Statewide tornado counts.

Counties with more people also tend to have more tornado reports. This is particularly true for Mississippi which has a correlation between tornado frequency and population of.49 [(.34, .62) 90% CI] and for South Dakota which has a correlation of.39 [(.20, .55) 90% CI]. The pattern of tornadoes across Illinois features a diagonal axis of high frequency from southwest to northeast similar to the pattern noted in [41]. However, this axis coincides with larger and more densely populated counties compared to the state average. The pattern of tornadoes in Mississippi features a hot spot in the vicinity of the city of Jackson. Across Ohio, tornadoes are notably fewer in the mountainous regions of the southeast. Marginally significant downward trends in statewide tornado frequency are noted for South Dakota and Ohio (Fig 11). Since 2000, a slight increase in the number of tornadoes is noted in Illinois and Mississippi.

Population density is a significant term in each of the models with South Dakota having the largest effect showing a 28% increase in tornado reports for a doubling of the population. Mississippi is next with a 20% increase in tornado reports for a doubling of the population. Population is only marginal significant for Ohio, likely related to the relatively high population density of the state compared to the other states examined here. A significant downward trend at a rate of 1.7% per year is noted in the model for South Dakota tornadoes and a significant upward trend at a rate of 2.4% per year is noted in the model of Mississippi tornadoes. No significant upward trends are noted for tornadoes in Illinois and Ohio. The interaction term is significant for Mississippi and Ohio, marginally so for Illinois, and not significant for South Dakota.

**Fig 12:**
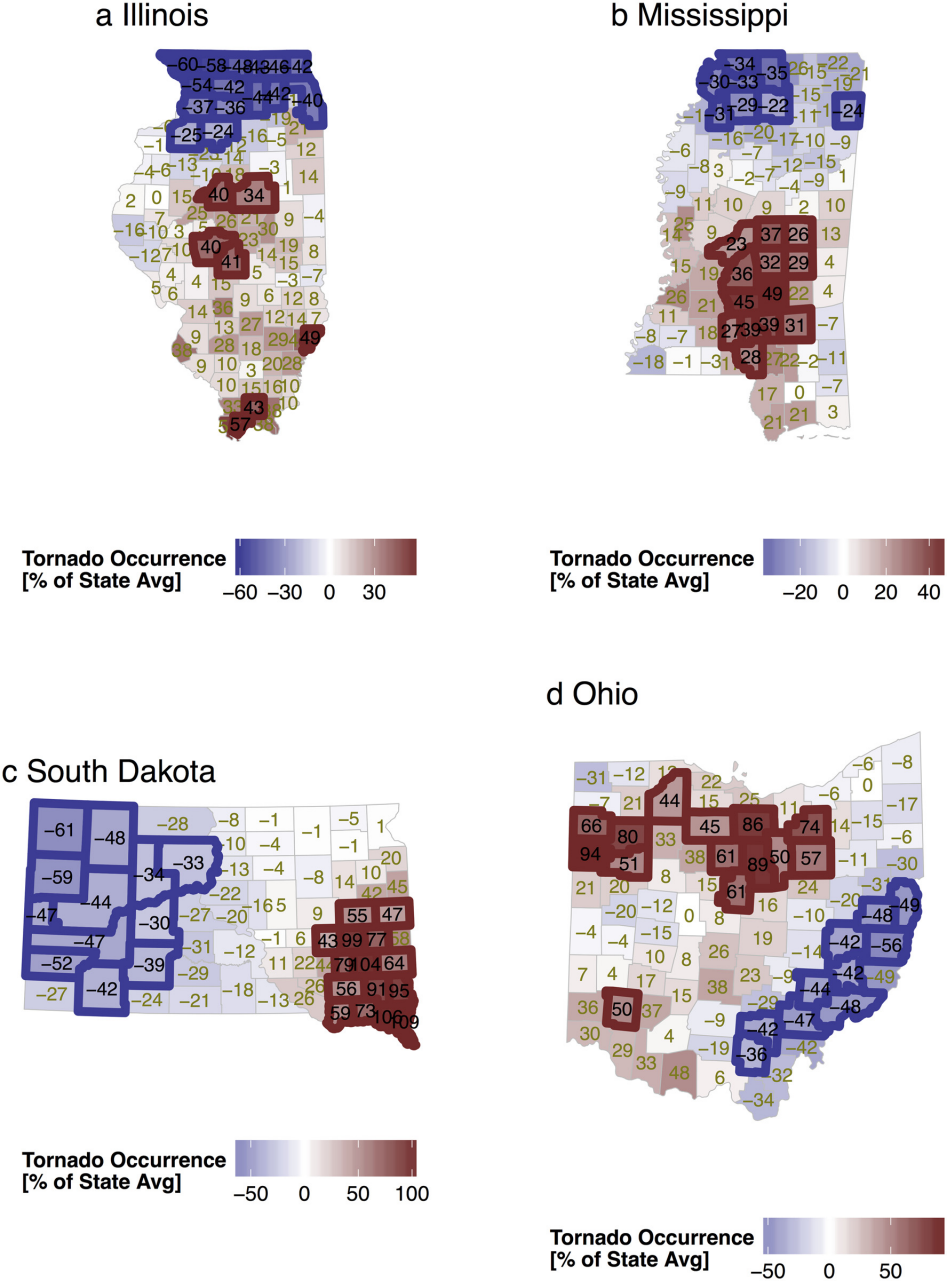
Correlated random effects from the state tornado models.

Maps showing the correlated random effects from the state models are shown in Fig 12. Illinois features a band of significantly below average frequency across the northern quarter of the state with much of the rest of the state above average. Some significant hot spots of above normal activity are noted across the midsection and over the extreme south. Mississippi shows a similar pattern with below normal frequency in the north and higher than average frequency across central and southern parts of the state. These north-south gradients are partially hidden in the map of raw counts but become conspicuous when controlling for county size and population density. The gradients are consistent with what would be expected over the long-term as the tornado season is longer in the south. South Dakota shows a well-defined mainly east-west gradient with significantly more tornadoes across the southeast and significantly fewer tornadoes in the west. Ohio features significantly fewer tornadoes across the southeast and a band of significantly more tornadoes running from near the city of Canton westward to the state line. The model with a correlated random-effects term is a type of smooth algorithm that accounts for population changes, differences in exposure, and trends.

**Fig 13:**
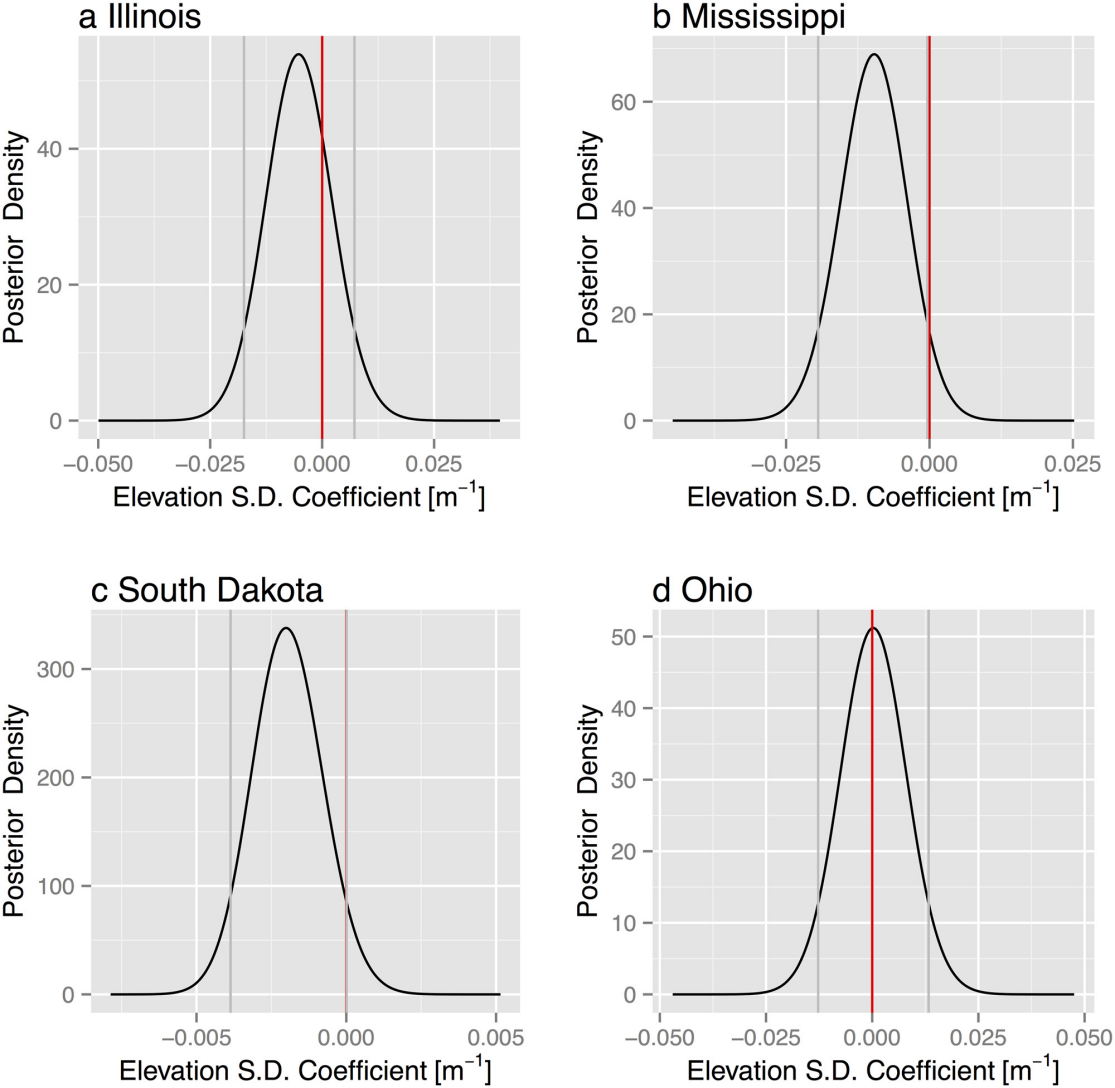
Posterior density of the elevation standard deviation term from the state tornado models.

Terrain roughness is a significant factor in the model for Mississippi tornadoes and marginally so for South Dakota but not elsewhere (Fig 13). The terrain roughness coefficient for all the states is negative indicating more tornadoes with smoother terrain. The magnitude of the effect is a 10% reduction in Mississippi tornadoes for every ten meter increase in elevation standard deviation and a 2% reduction in South Dakota tornadoes for the same amount of increase in roughness. County-level elevation standard deviations range from 2 to 35 m in Mississippi and from 6 to 420 m in South Dakota. The CWAs are not a significant factor in explaining the pattern of tornadoes in Illinois and Ohio. However, in Mississippi, the JAN CWA (Jackson, MS) has significantly more tornadoes (41%) than elsewhere in the state and the MOB CWA (Mobile, AL) has significantly fewer tornadoes (53%). In South Dakota, the FSD CWA (Sioux Fall, SD) has significantly more tornadoes (66%) than elsewhere in the state and the UNR CWA (Rapid City, SD) has significantly fewer tornadoes (34%). These difference, especially for South Dakota, likely reflect real differences in climatology rather than differences in warning and verification procedures. Our results are not directly comparable to studies of tornado risk that use multiple state areas and that use raw tornado reports without a correction for the bias associated with population [13].

### Summary and Future Directions

Tornadoes are discrete events, clustered in space and time, and locally quite rare. This makes it difficult to construct a regional climatology. Here a statistical model is demonstrated that overcomes some of these difficulties and that produces a smoothed regional-scale climatology of tornado occurrences. The model is applied to data aggregated to the county level. Data consist of annual population and tornado counts as well as an index of terrain roughness derived from a digital elevation model. The statistical model includes a term that represents the smoothed frequency relative to the state average after accounting for changes in reporting from population shifts and from improvements in rating procedures. The model is Bayesian and is fit using the method of integrated nested Laplacian approximation (INLA). A map of the correlated random-effects term shows where tornado activity is high relative to the state average. The model is used to assess whether high-resolution variation in terrain elevation is related to tornado frequency and whether there are differences in tornado activity by CWA.

The data preparation and model-fitting procedures are described using data from Kansas over the period 1970–2013. A key finding is that Kansas tornado reports increase by 13% with a two-fold increase in population but the influence of population density is decreasing. Independent of this relationship, it is found that tornadoes have been increasing at an annual rate of 1.9% perhaps related to an increasing number of storm chasers. Another key finding is the significant pattern of correlated residuals showing more Kansas tornadoes in a corridor of counties running roughly north to south across the west central part of the state. The model is improved by adding a term indexing terrain roughness. The magnitude of this effect, estimated by the posterior mean of the coefficient, amounts to an 18% reduction in the number of tornadoes for every ten meter increase in elevation standard deviation. The model shows tornadoes are 51% more likely to occur in counties served by the CWAs of DDC and GID compared with elsewhere in the state.

Flexibility of the model was illustrated by fitting it to data from other tornado-prone states including Illinois, Mississippi, South Dakota, and Ohio. Population changes are an important term especially in South Dakota and Mississippi. In Mississippi, the model indicates a 20% increase in tornado reports for a doubling of the population. A significant downward trend at a rate of 1.7% per year is noted in the South Dakota tornado model and a significant upward trend at a rate of 2.4% per year is noted in the Mississippi tornado model. The Brier score is lowest for the Ohio model.

Terrain roughness is a significant explanatory factor for Mississippi tornadoes and a marginally significant factor for South Dakota tornadoes, but the term is negative in the models for the other states considered. Across Mississippi, the magnitude of the roughness effect amounts to a 10% reduction in tornadoes for every ten meter increase in elevation standard deviation. The CWAs are not a significant factor in explaining the pattern of tornadoes in Illinois and Ohio. However, in Mississippi, the Jackson CWA sees 41% more tornadoes on average than elsewhere in the state. In South Dakota, the Sioux Falls CWA sees 66% more tornadoes than elsewhere in the state. These spatial variations likely reflect real differences in tornado climatology rather than differences in warning and verification procedures.

Future studies will test additional hypotheses. For example, is the influence of roughness strongest for the weakest tornadoes? The model could be extended to include other local and regional variables such as land use and land cover. For example, since forests may reduce inflow into developing storms, are tornadoes less likely over forested regions? Of particular interest is a test of the physical hypothesis that gradients in soil moisture contribute to tornado genesis [42]. Interest also centers on using the model-adjusted tornado counts as the actual risk of tornadoes together with demographic and social data to examine regions most vulnerable to tornadoes. The model can be extended to include multiple states and it can be adapted for use with a regular grid. The model can also be adjusted for other tornado data. For example, it might be interesting to use tornado path length as the response variable rather than tornado count. Path length provides a better metric for the influence a tornado has on a region [43].
